# Statement of Retraction: MicroRNA-146 attenuates lipopolysaccharide induced ovarian dysfunction by inhibiting the TLR4/NF- κB signaling pathway

**DOI:** 10.1080/21655979.2026.2730049

**Published:** 2026-10-01

We, the Editors and Publisher of the journal *Bioengineered*, have retracted the following article from publication.

He, F., Liu, Y., Li, T., Ma, Q., Yongmei, Z., He, P., & Xiong, C. (2022). MicroRNA-146 attenuates lipopolysaccharide induced ovarian dysfunction by inhibiting the TLR4/NF- κB signaling pathway. *Bioengineered*, 13(5), 11611–11623. https://doi.org/10.1080/21655979.2022.2070584

Since publication, significant concerns have been raised by a third party about the integrity of the data and reported results in the article. Specifically, concerns were raised about alleged image duplication with similar images from unrelated articles:

- Figure 2d, all panels appear to be duplicated with similar images in Figure 8, bottom row panels, from An et al, 2018 (https://doi.org/10.1155/2018/7163057)

- Figure 4, western blots appear to be duplicated with similar images in Figure 5B from Zhu et al, 2020 (https://doi.org/10.1590/1414-431x20209346)

Furthermore, internal integrity audits revealed concerns around authorship changes which were made between submission and acceptance of the article.

When approached for an explanation, the authors provided their original data and answered our queries, but these do not sufficiently address the concerns.

As verifying the validity of published work and determining authorship and contributions are core to the integrity of the scholarly record, we are therefore retracting the article. The authors have been informed of this decision.

We have been informed in our decision-making by our policy on publishing ethics and integrity and COPE guidelines. 

The retracted article will remain online to maintain the scholarly record and will be digitally watermarked on each page as ‘Retracted’. 

